# Feasibility of EBUS-TBNA for the molecular characterization of non-small cell lung cancer

**DOI:** 10.36416/1806-3756/e20230193

**Published:** 2024-05-08

**Authors:** Luis Vaz Rodrigues, Marta Viegas, Rosa Cordovilla, Luis Taborda-Barata, Vitor Sousa

**Affiliations:** 1. Serviço de Pneumologia, Instituto Português de Oncologia, Francisco Gentil, Coimbra, Portugal.; 2. Faculdade de Ciências da Saúde, Universidade da Beira Interior, Covilhã, Portugal.; 3. Serviço de Anatomia Patológica, Laboratório de Patologia Molecular, Instituto Português de Oncologia, Francisco Gentil, Coimbra, Portugal.; 4. Serviço de Pneumologia, Hospital Universitário de Salamanca, Salamanca, Espanha.; 5. Health Sciences Research Centre and UBIAir - CICS-UBI - Clinical & Experimental Lung Centre, Universidade da Beira Interior, Covilhã, Portugal.; 6. Instituto de Anatomia Patológica e Patologia Molecular, Faculdade de Medicina da Universidade de Coimbra, Coimbra, Portugal.; 7. Research Center for Environment, Genetics and Oncobiology - CIMAGO -Faculdade de Medicina, Universidade de Coimbra, Coimbra, Portugal.; 8. Centro de Pneumologia, Faculdade de Medicina, Universidade de Coimbra, Coimbra, Portugal.; 9. Serviço de Anatomia Patológica, Hospitais da Universidade de Coimbra, Coimbra, Portugal.

## TO THE EDITOR:

The accurate diagnosis of lung cancer (LC) relies on histopathological classification (HC) and molecular characterization (MC) for targeted therapies.^1^ Clinicians that deal with LC face the dilemma of how to apply minimally invasive interventions that yield large and well-preserved samples suitable for the demands of the histopathologist and the molecular geneticist.

A milestone in this pathway has been achieved with the introduction of EBUS-TBNA, which is currently the first-choice procedure for mediastinal staging of LC.^2^ ^,^ ^3^ On the other hand, MC of non-small cell LC (NSCLC) is a growing field of research with diverse strategies and heterogenous results.^4^ ^-^ ^7^

In this study we aimed to evaluate the current clinical practice of a large oncology referral centre concerning the feasibility of EBUS-TBNA-derived samples for MC of NSCLC.

We conducted a retrospective analysis (between January of 2019 and December of 2021) of all patients who underwent EBUS-TBNA for diagnosis and/or staging of NSCLC whose samples proceeded to MC. EBUS-TBNA was performed under general anesthesia with a BF-UC180F endoscope (Olympus, Tokyo, Japan) and 21G needles (ViziShot 2; Olympus). Samples were stored in formaldehyde and were processed as cell blocks for HC. *EGFR* status was determined by real-time polymerase chain reaction. If samples were negative, determination of *ALK* gene rearrangements by fluorescence in-situ hybridization followed.

Procedure and patient-related factors affecting sample adequacy were assessed. Finally, a timeframe was estimated from the initial endoscopic procedure and the final MC.

Descriptive and inferential statistical analysis was performed using the IBM SPSS Statistics software package, version 27 (IBM Corporation, Armonk, NY, USA). A logistic regression model was attempted to ascertain the presence of factors influencing MC results.

A total of 718 patients were subjected to EBUS-TBNA. Of these, 59 (8.2%) proceeded to MC, but only 38 (5.3%) had their MC performed in EBUS-TBNA samples. In the remaining 19 patients, MC was performed in other samples (6 in surgical specimens; 5 in bronchoscopy forceps biopsies; 4 in transthoracic CT-guided biopsies; and 4 in peripheral blood samples).

The patients included (N = 38) were mainly male (n = 25; 65.7%) with a median age of 67 years (range: 40-86 years). Nearly half had a relevant smoking history (12 former smokers and 8 current smokers). Most NSCLC were adenocarcinomas (n = 33; 86.8%), 3 were squamous cell carcinomas (SCC), and 2 were mixed adenocarcinoma and SCC (Table 1). All presented with locally advanced (stage IIIA, in 4; IIIB, in 6; and IIIC, in 3) or metastatic disease (IVA in 12; and IVB in 13). Programmed death-ligand 1 (PD-L1) status was ascertained in all patients and proved to be positive in 44.7% (in 2 patients with SCC and in 15 patients with adenocarcinoma), indeterminate in 5.2% (mixed adenocarcinoma and SCC, in 1; and adenocarcinoma, in 1), and negative in the remainder 50%.

**Table 1:** Histopathological classification and mutational profiling of the sample of patients with non-small cell lung cancer (N = 38).

Histopathological classification	Mutational profiling	n (%)
Adenocarcinoma	Non-mutated	26 (68)
	*EGFR* mutated	5 (13)
	*ALK* rearrangements	2 (5)
Squamous cell carcinoma	Non-mutated	1 (3)
	*EGFR* mutated	1 (3)
	*ALK* rearrangements	1 (3)
Mixed squamous cell carcinoma	Non-mutated	1 (3)
	*EGFR* mutated	1 (3)

A median of 2 lymph node stations were approached per patient (range: 1-4), with a median number of 3 needle passes (range: 3-8) per lymph node.

Overall, 34 out of the initial 38 cases (89.5%) were satisfactory for *EGFR* mutation testing, whereas 26 out of 32 (81.3%) were suitable for *ALK* rearrangement testing. Clinically relevant *EGFR* mutations were found in 6 patients (15.7%). *ALK* rearrangements were found in 2 cases (Table 1).

Mutated patients were mainly non-smoker males (5 out of 8) and presented with metastatic disease (stage IVB, in 4; and IVA, in 3).

The median time between EBUS-TBNA sampling and final MC was 20 days (range: 7-590 days). An in-depth analysis of this measure showed 11 cases (>30 group) in which this timeframe surpassed 30 days (median: 186 days; range: 48-590 days), whereas that was below 30 days in the ≤30 group (median: 18 days; range: 7-30 days). The patients in the >30 group were mainly in stage III (7 out of 11) whereas those in the ≤30 group were mainly in stage IV (20 out of 27).

MC was pivotal in determining the therapeutic options. All mutated patients were referred for targeted therapy (anti-EGFR, in 6; and anti-ALK, in 2). Immunotherapy was offered as frontline therapy in 15 non-mutated, PD-L1 positive patients. Platinum-based chemotherapy was the option for 15 additional patients. Best supportive care was offered to 2 patients that suffered severe performance status deterioration (ECOG 3 and 4) throughout the course of diagnosis and staging.

Due to the small sample size, a logistic regression model could not be built to ascertain factors associated with the feasibility of EBUS-TBNA for MC. Nevertheless, the 4 cases whose samples were insufficient belonged mainly to the puncture of a single (in 3 cases) or dual (in 1 case) lymph node stations with a median of 4 needle passes per lymph node station (range: 3-8).

This study unveils a clear underutilization of EBUS-TBNA (5.3%) for the MC of NSCLC. Various reasons may account for this. First, NSCLC staging was determinant, since only candidates for systemic therapy (stages III or IV) were referred for MC. Secondly, there was preferential utilization of other biological samples that were perhaps considered more cell enriched, such as surgical or CT-guided biopsies. An intriguing finding of the study was the option for peripheral blood sampling, in 4 cases. The wider accessibility of peripheral blood samples may offer an explanation, but it is still surprising that this material was preferred over EBUS-TBNA samples.

As previously reported,^6^ ^,^ ^7^ EBUS-TBNA was feasible for MC in most cases (89.5% for *EGFR* and 81.3% for *ALK*). Also, in agreement with previous reports,^6^ ^,^ ^7^ *EGFR* analysis outperformed *ALK*. The sequential method applied in this study may offer an explanation. Samples were sequentially used for HC, *EGFR* testing, and only afterwards released for *ALK* testing, which means that only largely cellular samples could suffice all processes.

Two patient groups were identified based on the timeframe of MC. The >30 group mainly included patients with less advanced disease stages who probably undergone multimodal therapeutic strategies in which systemic therapy was likely delayed. In contrast, the ≤30 group included patients with metastatic disease in which systemic therapies came first and hence the prompter need for an up-front MC.

Our study showed a relatively low prevalence of mutations (Table 1). We observed 18% of mutated adenocarcinomas (*EGFR* mutations, in 13%; and *ALK* rearrangements, in 5%), and there was only 1 case of *EGFR*-mutated SCC (3%), as was there 1 case of *EGFR*-mutated mixed SCC and adenocarcinoma (3%), which agrees with publications reflecting Western populations.^8^ ^,^ ^9^

When assessing factors that could influence the feasibility of molecular profiling EBUS-TBNA samples, we could not safely establish statistically significant relationships. Nevertheless, we observed a trend toward lower yields in patients with a smaller number of lymph nodes approached despite a seemingly higher number of needle passes in these cases. This perhaps reflects the clinical perception of a lower probability of achieving a complete diagnosis in cases when just one lymph node station was approached.

In conclusion, our study highlights the value EBUS-TBNA on obtaining sufficient samples for MC of NSCLC. Nevertheless, questions are raised about sequential approaches and the time required for molecular results, for which additional studies, namely addressing the added value of multiplex simultaneous analysis, are still warranted.^6^ ^,^ ^10^
